# Using the eSexual Health Clinic to access chlamydia treatment and care via the internet: a qualitative interview study

**DOI:** 10.1136/sextrans-2017-053227

**Published:** 2017-10-07

**Authors:** Catherine R H Aicken, Lorna J Sutcliffe, Jo Gibbs, Laura J Tickle, Kate Hone, Emma M Harding-Esch, Catherine H Mercer, Pam Sonnenberg, S Tariq Sadiq, Claudia S Estcourt, Maryam Shahmanesh

**Affiliations:** 1 Research Department of Infection and Population Health, Centre for Sexual Health and HIV Research, Institute for Global Health, University College London, London, UK; 2 Barts and the London School of Medicine and Dentistry, Blizard Institute, Centre for Immunology and Infectious Diseases, Queen Mary University of London, London, UK; 3 College of Engineering, Design and Physical Sciences, Brunel University, London, UK; 4 Applied Diagnostic Research and Evaluation Unit, Institute for Infection and Immunity, St George’s University of London, London, UK; 5 HIV/STI Department, National Infection Service, Public Health England, London, UK; 6 School of Health and Life Sciences, Glasgow Caledonian University, Glasgow, UK

**Keywords:** chlamydia infection, sexual health, communication technologies, qualitative research, compex interventions

## Abstract

**Objective:**

We developed the eSexual Health Clinic (eSHC), an innovative, complex clinical and public health intervention, embedded within a specialist sexual health service. Patients with genital chlamydia access their results online and are offered medical management via an automated online clinical consultation, leading to antibiotic collection from community pharmacy. A telephone helpline, staffed by Sexual Health Advisers, is available to support patients and direct them to conventional services if appropriate. We sought to understand how patients used this ehealth intervention.

**Methods:**

Within exploratory studies of the eSHC (2014–2015), we conducted in-depth interviews with a purposive sample of 36 patients diagnosed with chlamydia, who had chosen to use the eSHC (age 18–35, 20 female, 16 male). Thematic analysis was conducted.

**Results:**

Participants described choosing to use this ehealth intervention to obtain treatment rapidly, conveniently and privately, within busy lifestyles that hindered clinic access. They described completing the online consultation promptly, discreetly and with ease. The information provided online was considered comprehensive, reassuring and helpful, but some overlooked it in their haste to obtain treatment. Participants generally described being able to collect treatment from pharmacies discreetly and promptly, but for some, poor awareness of the eSHC by pharmacy staff undermined their ability to do this. Those unsuitable for remote management, who were directed to clinic, described frustration and concern about health implications and clinic attendance. However, the helpline was a highly valued source of information, assistance and support.

**Conclusion:**

The eSHC is a promising adjunct to traditional care. Its users have high expectations for convenience, speed and privacy, which may be compromised when transitioning from online to face-to-face elements of the eSHC. Managing expectations and improving implementation of the pharmacy process, could improve their experiences. Positive views on the helpline provide further support for embedding this ehealth intervention within a specialist clinical service.

## Introduction

STI rates remain high in England, despite existing STI control measures.^1 2^ Prompt effective treatment of diagnosed STIs is vital to reduce harms associated with long-term infection and onward transmission. However, timely access to genitourinary medicine (GUM) clinics is threatened by increasing financial pressures.^3 4^ ehealth may increase access and convenience, at a potentially reduced cost.^5–7^ Globally, the push for internet-based healthcare, combined with the realisation that traditional models of face-to-face physician-led care are unsustainable, has never been stronger, but underpinning research on acceptability and effectiveness is lacking.

Sexual health is a promising arena for ehealth. In the UK, young people, a risk-group for STI,^1^ have near-universal internet access^8^ and report greater internet-use for help/advice with their sex-lives than older age-groups.^9^ Online services may enhance privacy in this sensitive and stigmatised area.^10^ However, development and evaluation of ehealth services, as complex interventions, requires an understanding of the mechanisms and contexts in which they work,^11^ including a contextualised understanding of users’ behaviour.^12^

**Figure 1:**
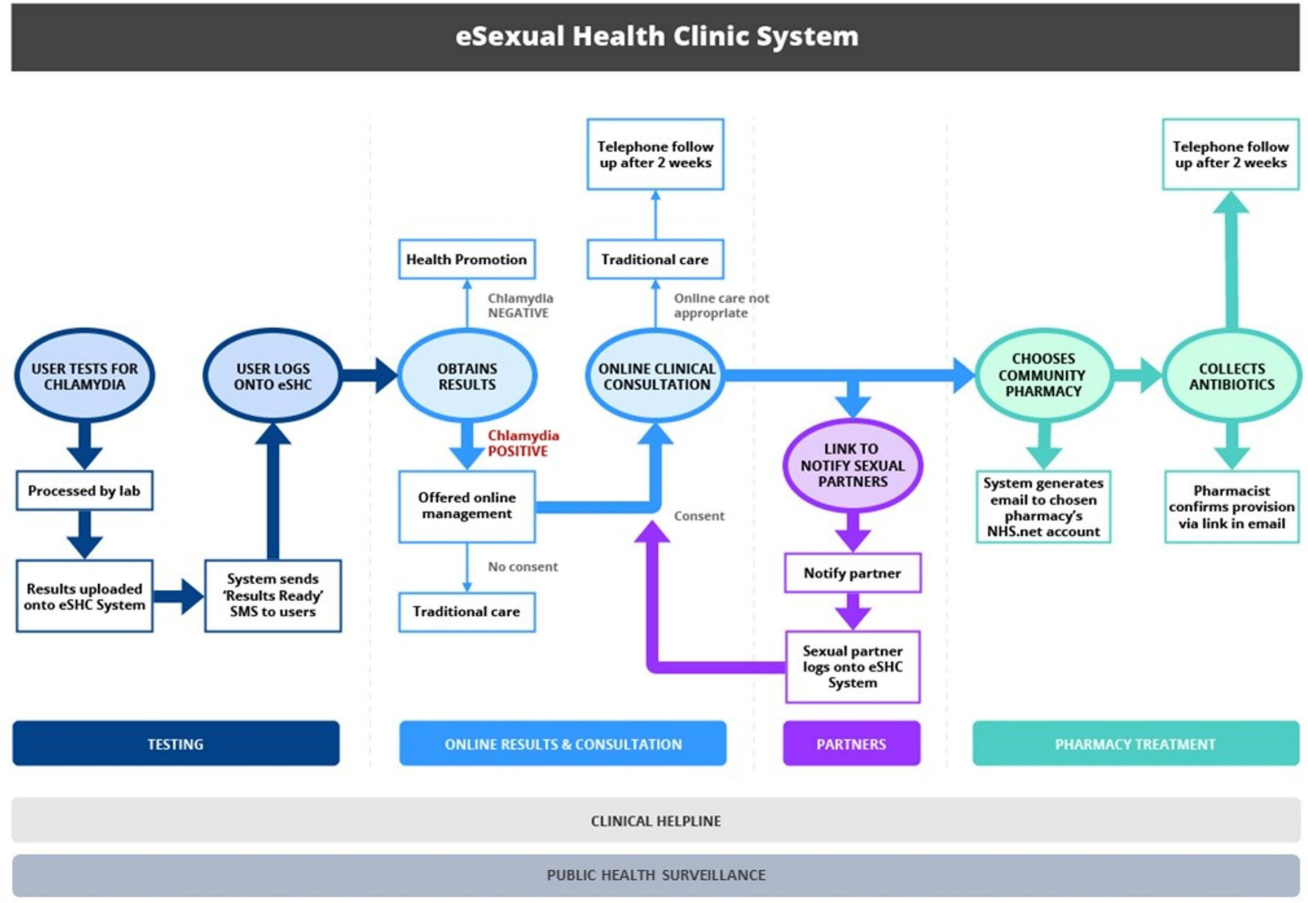
The eSHC. This figure was published in The Lancet Public Health, 2017;2(4):182–90, Estcourt *et al*, ‘The eSexual Health Clinic system for management, prevention and control of sexually transmitted infections: exploratory studies in people testing for *Chlamydia trachomatis*.’, Elsevier 2017. Notes: Only those testing chlamydia positive were included in the current study. Those testing negative were not interviewed and we do not report data on the small number of people who participated as notified sexual partners of chlamydia-positive study participants. Health information was available on results screen and via links to reputable websites. Patients who reported, in the online consultation, symptoms indicative of potentially complex infection or allergies, an underlying medical condition or that they were on medication which meant that they needed an alternative antibiotic, were alerted to telephone the helpline to facilitate access to traditional care. The Sexual Health Adviser staffing the helpline was simultaneously alerted to telephone the patient, in case they did not make contact. All those consenting to participation in the Exploratory Studies were followed up (top of figure). eSHC, eSexual Health Clinic; SMS, short messaging system (text message).

Through detailed formative research,^10 13 14^ we developed an online clinical pathway for STI management, using genital chlamydia as an exemplar. This pathway was deployed within an eSexual Health Clinic (eSHC, figure 1),^15^ a web-application, which people logged into to access their STI test results. Via the eSHC web-application, people testing positive for chlamydia were provided with information and were offered the opportunity to follow an automated online clinical consultation, consisting of tailored questions on presence of symptoms, medical history, drugs and allergies, sexual history and a risk assessment for blood borne viruses.^16^ If safe and appropriate, this led to collection of treatment from a chosen community pharmacy. A helpline, staffed by a specialist Sexual Health Adviser (SHA), was available throughout and facilitated access to clinic/general practice (GP) for those for whom ‘remote’ management (away from clinical services and medical professionals) was inappropriate. All users were followed up by telephone by an SHA, to check treatment was taken correctly, ascertain partner notification (PN) outcomes and provide support if needed.

The eSHC is unique within the National Health Service (NHS), in enabling users to receive a new medical diagnosis online and (if safe, appropriate and desired) proceed to treatment, ‘remote’ from medical settings and with minimal supervision. The few existing NHS online STI services enable access to testing,^17 18^ but treatment of those testing positive requires individualised assessment by a clinician,^16^ in healthcare settings or by telephone.

We piloted the eSHC in exploratory studies.^15^ Within these, we conducted qualitative research among people who had tested positive for chlamydia (the focus of this article) to understand the experience of using this internet-based intervention, in order to inform its refinement and future evaluation.

## Methods

### Setting and population

This qualitative study took place among participants of the exploratory studies who had tested positive for genital chlamydia. These studies’ methods are detailed elsewhere.^15^ Briefly, people who had tested in two GUM services or via South London internet-based postal home-sampling (‘Checkurself’, within the National Chlamydia Screening Programme, NCSP) received their chlamydia-positive result online and were offered the opportunity to use the eSHC (figure 1). Those coinfected with another STI or extragenital chlamydia, aged under 16, unable to read English or not providing a mobile phone number, were ineligible and managed as per standard care.

### Interview recruitment and sampling

During the eSHC’s follow-up phone-call, SHAs asked patients with adequate spoken English for permission to pass their first name and mobile number to a researcher, to discuss a possible telephone interview.

Sampling was purposive,^19^ with quotas of 6–12 women and men in age-group (16–24, ≥25 years) and testing service (clinic or ‘Checkurself’) categories (total: eight categories). Additional sampling categories, developed during data collection, captured diversity in eSHC use (which was unknown a priori).

### Data collection

One female interviewer (CA) conducted in-depth interviews by telephone, with oral informed consent. She introduced herself as a non-clinical researcher, interested in understanding what it is like to use the eSHC. Interviews took place on average 5 days after completion of eSHC follow-up, using a topic guide informed by previous research^10 13^ (see online supplementary file 1). Participants were offered a £30 e-voucher as reimbursement. Data collection ceased when sampling quotas were full and no new findings were emerging.

10.1136/sextrans-2017-053227.supp1 Supplementary file 1 s1

### Data management and analysis

Transcripts of audio-recordings (average: 44 min) were checked to ensure accuracy and anonymity.

We conducted a thematic analysis,^20^ using Framework^21^ for data management, in which data are organised into matrices by participant and descriptive code. Codes were based on elements of the eSHC (figure 1) and topics identified in previous research.^10^ Two researchers double-coded a selection of transcripts and agreed with the codes (CA and LS, in discussion with MS). CA coded transcripts in NVivo and then identified four emergent themes describing how participants used the eSHC from these ordered data. Themes were refined in discussion with LS and MS and applied to the data in a second phase of coding.

Ethical approval was granted by Brighton & Sussex (NHS) Research Ethics Committee (ref:13/LO/1111).

## Results

Sixty-two per cent (87/140) of the eligible patients agreed to be contacted, of which the interviewer attempted to contact 58 and interviewed 40 (69%^i^, including four partners of chlamydia-positive eSHC users, data not reported). Table 1 describes this study’s 36 participants.

**Table 1:** Sample characteristics, reported behaviours and experiences

Gender*			Women	Men	Total
**Demographics**					
Age (years)*	18–24		10	8	18
	25–35		10	8	18
Ethnicity†	Asian		1	2	3
	Black		2	5	7
	Mixed		3	1	4
	White		14	8	22
Relationship status†	Single		7	9	16
	In relationship		8	3	11
	Split up with partner, related to chlamydia diagnosis		4	0	4
	Casual partner/s		1	3	4
	Not discussed		0	1	1
Sexual orientation†	Heterosexual, straight		18	16	34
	Not discussed (but recent partners opposite sex)		2	0	2
**Experience of sexual healthcare**					
Previous STI testing†	Yes		16	12	28
	No		3	4	7
	Not discussed		1	0	1
Previous STI diagnosis†	Yes		7	5	12
	Not had chlamydia before		1	0	1
	No		12	11	25
Testing (this episode)*	In a sexual health (GUM) clinic		12	8	20
	Via internet-based postal home-sampling (NCSP Checkurself)		8	8	16
**eSexual Health Clinic use**					
Route to treatment*	Directed to clinic/GP		6	1	7
	Disengaged from eSHC and treated in clinic		2	1	3
	Completed to pharmacy treatment collection		12	14	26
	of which:	Problems with treatment collection: 2+ trips to pharmacy and/or helpline use	3	1	4
		No problems at pharmacy or problems resolved during one visit without helpline	9	13	22
Helpline use	Yes, self-initiated		6	0	6
	Yes, when prompted, re: being directed to clinic		3	1	4
	No		11	15	26
**Total**			20	16	36

*Primary sampling characteristics.

†Secondary sampling characteristics, by which we sought diversity across the entire sample.

GUM, genitourinary medicine; NCSP, National Chlamydia Screening Programme.

### Themes describing use of the eSexual Health Clinic

#### 1: Do something, fast!

Participants assumed that the eSHC would facilitate rapid treatment, which influenced their choice to proceed online following their chlamydia diagnosis. As this participant explained:

*[the website] gives you the options, you know, go and see someone or go online. And I thought, well actually, y’know, if I wanna get treated now…* (26-year-old man, tested via Checkurself)

This urge to act quickly following receipt of their chlamydia diagnosis led to some participants to proceed immediately online using their smartphones. They described how this enabled rapid completion of the online consultation, while maintaining privacy (discussed below, theme 2), even in public locations:

*I wanted to get it sorted straight away[…] And mobile’s quite discreet[…] I thought for all everyone around me knows I was just on facebook.* (29-year-old man, tested in clinic, completed online consultation at his desk in a shared office)

For others, feelings of urgency were balanced with privacy concerns and technology constraints (themes 2 and 3). Thus, some completed the online consultation later the same day they received their results and changed location and/or device.

*[During my lunch-break] I just tried to find some privacy and to go and check the information again on the laptop first, because, well I had more time, and you have a bigger screen so it’s just easier to read. So I really read all the information which was included in the results and, yeah, after, I answered the questions and ordered the medication online.* (26-year-old man, tested in clinic)

Participants typically described the information provided online as helpful and comprehensive. However, some of those who felt panic and rushed through the consultation on their smartphones while in public, considered the information inadequate and mentioned missing details which were actually present. As this man, who described lacking information about what chlamydia is, explained:

*Maybe it did say that but I was too busy frantically trying to [laughs] get to the antibiotic stage.* (29-year-old man, tested in clinic)

In contrast, using the eSHC’s web-interface at relaxed pace, in greater privacy (theme 2) appeared to result in greater uptake or recall of the information provided.

Participants described the process of completing the online clinical consultation and selecting (online) a pharmacy from which to collect treatment, as quick and easy. Treatment collection from pharmacies generally worked well, preserving participants’ desire for prompt treatment access and comparing favourably with their experiences of clinic. Describing the process as ‘*seamless*’, this man explained that pharmacy staff:

*…seemed to know exactly what I was here for and I said I was part of an eSTI trial, grabbed some medicine, and I was out within about five minutes.* (22-year-old, tested in clinic)

However, in some cases, pharmacy staff were apparently unaware of the study or could not locate treatment packs, such that participants needed to return to the pharmacy on another occasion. This led to a short delay for participants (a few days), but had a significant impact on their experience, in the context of having an STI requiring treatment:

*… it just seemed like the longest wait ever and I was quite frustrated at the time, quite upset.* (26-year-old woman, tested in clinic)

Participants generally described taking treatment the day they collected it (and with a good understanding of information received in treatment-packs and online.)

#### 2: Protecting privacy

All participants described acting to conceal their STI and treatment-seeking from those around them, but to varying extents. Some, particularly Checkurself users, sought to avoid the embarrassment and exposure that they associated with sexual health clinic attendance. For them, the eSHC was:

*…definitely a much more, sort of less embarrassing way to go about it, without, y’know, having to worry about seeing anyone you know [in clinic]*. (20-year-old woman, tested via Checkurself)

When completing the online consultation, some protected their privacy by using their smartphone, while others changed location (as discussed, theme 1). Participants described providing information via the online consultation with ease and some considered it a more private way of providing sexual history details, with:

*…no one there to give you their opinions straight away, or even kind of make a gesture that would suggest their opinion. You can be as honest as possible, I think. You can be more honest than if you go to a clinic.* (27-year-old man, tested in clinic)

While some participants mentioned concerns about online data security, they appeared to accept this as an inevitable part of the online experience:

*…on the internet, it’s just that fear of maybe someone else is going to get the information. [Interviewer: Was that a concern for you?] No, no, er- no, actually cos I do a lot of things on the internet, so I actually trust the internet. A lot.* (22-year-old man, tested via Checkurself)

However, privacy was sometimes threatened during transitions from online, to offline, public space. For instance, when the pharmacy treatment collection process worked as intended, participants could maintain discretion about their reason for attending the pharmacy, but when pharmacy staff were unaware of the study, participants’ attempts to explain their needs in this public setting were perceived to compromise privacy:

*…three or four people sat about a metre behind me[…] I don’t think [staff] clicked that it was something I didn’t really want to be shouting about. [They said:] ‘No I don’t get– I don’t know what you’re on about!’ Erm, just shhh…* (24-year-old woman, tested via Checkurself)

#### 3: Choices and non-choices

Positive perceptions of the eSHC as a fast, private way to obtain treatment influenced participants’ choice to use it (themes 1 and 2). In addition, they described how this choice was influenced or constrained by difficulties (re)attending conventional services, in the context of busy lifestyles.

*[To attend clinic] I have to either book an appointment, which is also not gonna be easy cos of my working hours, or get there really, really early[…] when I saw it, an online option to do it, I thought this is much— probably gonna be much easier.* (27-year-old man, tested in clinic)

Certain constraints also influenced how participants used the intervention. For instance, although all described completing the online consultation the day they received their results (theme 1), some delayed collecting treatment because they were away from home (a constraint which also hindered their access to conventional services via which they could obtain treatment, such as sexual health clinics or general practice).

Problems that some participants experienced with pharmacy treatment collection were exacerbated when participants faced difficulties reattending:

*…they were asking me to come back another day and I was like, I can’t do that[…] I already leave work earlier to make sure I can get my treatment, and like they won’t allow me like to leave earlier every day…* (27-year-old woman, tested in clinic)

#### 4: Seeking peace of mind

Following diagnosis, the prospect of a quick, discreet and convenient route to treatment via the eSHC (as discussed, themes 1–3) was reassuring, as was the eSHC’s basis in NHS services, which conferred trustworthiness:

*…I knew that the [home sampling-]kit was from the NHS. I, I just trusted everything that came with it, so I trusted the text, the link, and my results. I also trusted the treatment.* (21-year-old woman, tested via Checkurself)

To resolve concerns about their chlamydia infection, its treatment and implications, participants sought information online, some used the helpline and two described contacting other services (eg, GP). In the interviews, participants typically discussed how it is ‘*definitely*’ necessary to have a helpline available. However, while they were using the eSHC, some had not noticed that a helpline was provided when they were using the eSHC (despite the number being displayed on each page of the eSHC web-application; they commented that they probably had not noticed or looked for it, because they had not needed it themselves). Helpline users described using it for information, technical assistance and/or support:

*I probably knew what to do, but it’s just because I was a bit overwhelmed about everything. I thought I need to speak to someone…* (32-year-old woman, tested via Checkurself)

Those who sought support described the helpline particularly positively:

*…it’s always nice to have someone to kind of look after you and make sure that everything is fine.* (26-year-old woman, tested in clinic)

Similarly, participants appreciated the *‘closure*’ and ‘*personal touch*’ (29-year-old man, tested in clinic) of the follow-up phone-call:

*…if no one called me, then I would’ve felt a bit like, ‘well, is it done, what should I do?’* (22-year-old woman, tested via Checkurself)

### Alternative experiences

We used alternative experiences described by participants to refine themes and to illustrate further how they interrelate.^22^

#### Being directed to clinic for treatment

As an integral part of the eSHC, patients whose online consultation responses indicated that ‘remote’ provision of Azithromycin was inappropriate were instructed to call the helpline and could not continue online. By telephone, the SHA emphasised the importance of attending clinic, offered to book an appointment and provided information.

Those who had disclosed symptoms online described annoyance and anxiety about their health and about attending clinic—which, by choosing the eSHC, many had sought to avoid (see themes 1–3). For instance, this woman felt *‘really upset*’, because she *‘thought it would be a bit embarrassing to go to the clinic*’:

*… also because it said [online], ‘because you said that you’ve got one of the symptoms you need to come,’ so I was like, I hope it doesn’t mean it’s going to be more complicated…* (22-year-old, tested via Checkurself)

Helpline contact, informing participants of the precautionary nature of this visit, was reassuring (theme 4). However, some remained unconvinced that clinic attendance had been necessary.

#### Abandoning the eSHC

Two participants received their diagnosis online, but abandoned the eSHC and attended clinic. Both described being particularly upset about the impact of their diagnosis on relationships (and one, on her health). Contrasting with the busy schedules discussed by others, both described having the flexibility to attend clinic the day they received their results (themes 1, 3) and sought reassurance through human contact (theme 4).

*I felt more relieved, like, talking to someone[…] even though I knew, you know, I had all the information[…] I was looking for a bit of comfort.* (34-year-old woman, tested in clinic)

## Discussion

This is the first qualitative study describing the experience of using a novel online sexual health intervention, which enabled some users to proceed from receipt of results to treatment collection without seeing or speaking to a clinician.^15^ Generally, the eSHC enabled patients to receive chlamydia treatment promptly and discreetly, within busy lifestyles. They provided sensitive information online easily and without embarrassment, yet valued the helpline’s availability. Greatest satisfaction was expressed by those who obtained treatment from community pharmacies without problems, for whom the perceived benefits of online care were preserved ‘offline’. However, these benefits were sometimes compromised when transitioning from online, to offline/public spaces: among the minority^15^ directed to clinic for treatment or at pharmacy treatment collection.

The eSHC provides an alternative management option for patients with uncomplicated chlamydia and was embedded within a specialist service, providing safeguards, specialist health professional support and follow-up, and facilitated clinic access. Positive views about the eSHC helpline (staffed by sexual health clinic SHAs) support the eSHC’s basis in specialist services. Patients’ expectations of a rapid, discreet and convenient service must be borne in mind during refinement of ‘offline’ parts of the eSHC. Clarification that not everyone will be medically appropriate for online management may better manage expectations.

Awareness and uptake of online health information appeared to be influenced by context. Where users were calm and their surroundings private, they found the information comprehensive and reassuring. Our study highlights the impact of some users’ feelings of anxiety and urgency of treatment-seeking, on uptake of online health information following diagnosis of an acute, stigmatised condition. The potential loss of ‘teachable moments’^23^ that this precipitates may apply to future internet-based sexual health services, eg, for emergency horomonal contraception, or HIV self-testing. Despite evidence of effectiveness of some internet-based sexual health promotion interventions,^24^ these have not yet been studied within online care pathways. Further research is needed to explore ways to improve uptake of online health promotion, for those testing positive or negative, including consideration of ehealth literacy.

Despite participants being recently diagnosed with an STI, thus potentially difficult to research, we achieved a strong, diverse sample, qualitatively representing those who had tested in clinic and via internet-based home-sampling and those with/without experience of STI treatment, whose perspectives may differ. However, men who have sex with men (MSM) were unrepresented; very few participated in the exploratory studies (people with coinfection and extragenital chlamydia, both more common among MSM, were excluded).

All participants chose the eSHC, so our findings do not extend to everyone with chlamydia (or other STIs). Those with lower health literacy or digital literacy may be unable or unwilling to use ehealth.^25^ Patients were offered the eSHC after using established NHS testing services, which enhanced their confidence in using it.

Interviewing shortly after completion of care helped minimise recall issues. Telephone interviewing was appropriate to the sensitive topic and participants’ choice of ‘remote’ healthcare, but those who declined participation may have had higher requirements for privacy and convenience.

There is a dearth of similar studies. As e-prescribing is typically physician-mediated, studies of patients’ experience of this have limited relevance to the eSHC, while research on commercial online pharmacies’/vendors’ treatment provision focuses on quality and legality.^26–28^ Our findings extend and complement our previous research, which explored the acceptability of a hypothetical STI self-test and online care, in a younger population.^10^ Some differences (eg, lower concerns about online data security) may reflect the current study population’s older age-range and experience of internet-based healthcare.

This study informs the eSHC’s refinement for future evaluation. Mixed-methods analysis of the eSHC’s support for PN is underway. Future qualitative research must explore the views of non-users of the eSHC and MSM. Mindful of concerns that ehealth could widen health inequalities,^29 30^ evaluation must include assessment of the educational and socioeconomic status of users and non-users.

**Key messages**

- The eSexual Health Clinic is unique in supporting patients from online receipt of a new chlamydia diagnosis, to treatment, remotely and with minimal supervision.
- Building on formative research, we used qualitative interviews to generate a contextualised description of patients’ experience of using this novel ehealth intervention.
- Patients described obtaining treatment rapidly and discreetly online compared with attending a clinic, but valued optional access to specialist sexual healthcare professionals by telephone, for reassurance, assistance and information.
- Refinement to ‘offline’ parts of this ehealth intervention, to preserve privacy, convenience and speed of treatment, may further increase its acceptability.

## Footnotes

i Related to telephone recruitment, where phone calls sometimes were unanswered, reasons for non-participation were often unknown.
