# Supplementary material for: Using the eSexual Health Clinic to access chlamydia treatment and care via the internet: a qualitative interview study
Source: Sex Transm Infect. 2017 Oct 7;94(4):241–7. doi: 10.1136/sextrans-2017-053227 (PMC5969326; doi:10.1136/sextrans-2017-053227)
Supplement: Supplementary file 1 [file sextrans-2017-053227supp001.docx]

**Qualitative interview topic guide - summary**

**Opening questions/‘ice-breaker’:** Internet use – in general, for health

**First impressions of eSHC**

- Reason for choosing, expectations, awareness of other sources of treatment

**Interviewee talks through what happened:**

*Probe for details, views, reasons for actions taken, how interviewees worked out what to do at each stage, and to establish sequence of what happened & approximate timings.*

- Testing experience *(not asked of those participating as partners)*
- Reaction to result (or message from partner)
- Care pathway use – what happened
- Experience of providing information online (online ‘consultation’)

**[*if applicable:*] Being directed to clinic/GP**

- Reaction, what happened (helpline use? Attendance, receiving treatment)

**[*if applicable:*] Disengaging from the care pathway**

- Reasons, what happened

**Awareness and use of the telephone helpline**

**[*if applicable:*] E-prescription & picking up treatment from pharmacy**

- Selecting a pharmacy
- Picking up treatment
- Taking treatment
- Acceptability of this process

**What happened next?** Talk through to Clinical Follow-up telephone call

**[*if applicable:*] Partner notification***

- People to tell about chlamydia diagnosis? Managed to do this? Experience. Suggestions for making this easier?
- Awareness, understanding, use of link and code for partners
- Reasons why/why not used; in principle acceptability

***Topics to probe on unless already discussed:***

**Privacy**

- More or less private via eSHC compared to clinic/GP? Why?
- [*if privacy concerns* mentioned] What to keep private? Why?
- Comparison face-to-face vs. online sexual healthcare

**Support, information, the helpline and use of other services**

- Needs for info, support, help? At what stage? For what? What happened?
- Need for info/support now?
- Other health services used? What for/why/at what stage?
- Awareness, use, views of helpline (opening hrs?)

**Speed** – expectations & fit with what happened

**Previous experience of STI testing/diagnosis**

**Final words & recommendations**

- Expectations/needs met/unmet? Suggested improvements?
- How would you describe your experience? Recommend it? Why/why not?

**Thoughts on eSTI^2^ remote self-test**

**Reflections on interview; phone interview mode**

**Recorder off.** Collect demographic details, take email address. Thanks.

*After the interview: Interviewer makes field-notes on:*

*circumstances of recruitment and interview, including issues potentially affecting data quality/interpretation; candidate themes and saturation; refinements to interview technique and topic guide.*

*eSHC users’ experience with partner notification, and notified partners’ use of the eSHC, are not discussed in the current article.
